# Diet Modification before or during Pregnancy on Maternal and Foetal Outcomes in Rodent Models of Maternal Obesity

**DOI:** 10.3390/nu14102154

**Published:** 2022-05-21

**Authors:** Natassia Rodrigo, Sonia Saad, Carol Pollock, Sarah J. Glastras

**Affiliations:** 1 Department of Diabetes, Endocrinology and Metabolism, Royal North Shore Hospital, Sydney 2065, Australia; natassia.rodrigo@sydney.edu.au; 2 Kolling Institute of Medical Research, Sydney 2065, Australia; sonia.saad@sydney.edu.au (S.S.); carol.pollock@sydney.edu.au (C.P.); 3 Faculty of Medicine and Health, The University of Sydney, Sydney 2006, Australia; 4 Department of Renal Medicine, Royal North Shore Hospital, Sydney 2065, Australia

**Keywords:** high-fat-diet, programming, gestational, weight, kidney, liver, rodent, mouse

## Abstract

The obesity epidemic has serious implications for women of reproductive age; its rising incidence is associated not just with health implications for the mother but also has transgenerational ramifications for the offspring. Increased incidence of diabetes, cardiovascular disease, obesity, and kidney disease are seen in both the mothers and the offspring. Animal models, such as rodent studies, are fundamental to studying maternal obesity and its impact on maternal and offspring health, as human studies lack rigorous controlled experimental design. Furthermore, the short and prolific reproductive potential of rodents enables examination across multiple generations and facilitates the exploration of interventional strategies to mitigate the impact of maternal obesity, both before and during pregnancy. Given that obesity is a major public health concern, it is important to obtain a greater understanding of its pathophysiology and interaction with reproductive health, placental physiology, and foetal development. This narrative review focuses on the known effects of maternal obesity on the mother and the offspring, and the benefits of interventional strategies, including dietary intervention, before or during pregnancy on maternal and foetal outcomes. It further examines the contribution of rodent models of maternal obesity to elucidating pathophysiological pathways of disease development, as well as methods to reduce the impact of obesity on the mothers and the developing foetus. The translation of these findings into the human experience will also be discussed.

## 1. Introduction

The global incidence of obesity continues to rise at alarming rates. Concerningly, the incidence of women of childbearing age with obesity is escalating, leading to risks for both the mother and the child [1]. Obesity increases maternal complications, including gestational diabetes, miscarriage, hypertension, and pre-eclampsia during pregnancy [2]. It also predisposes children to develop obesity, hypertension, diabetes, cardiovascular disease, and chronic kidney disease later in life [3,4]. Combating this global spread of obesity is a major priority area for the World Health Organisation (WHO). Guidelines on the management of maternal obesity recommend that women with obesity attempt lifestyle modification prior to pregnancy to achieve a 5–10% body weight loss [5]. Despite this, there is scant evidence as to the benefit, with few studies assessing the clinical outcomes of preconception weight loss for women with obesity [6]. 

Animal models of maternal obesity are highly beneficial in probing the effects of maternal obesity on maternal and foetal outcomes, as well as interrogating the effectiveness of weight modulation during and prior to pregnancy. Animal studies have the advantage of controlled experimental design, allowing a specific maternal perturbation to be induced to determine its influence on maternal and perinatal outcomes while minimising the confounding effects of genetic and postnatal environmental influences. Further, rodent strains are highly susceptible to weight gain when fed a high-fat diet (HFD), and they can manifest features of the metabolic syndrome, namely obesity, insulin resistance, diabetes, and hepatic steatosis [7]. 

This article reviews the current evidence using rodent models of obesity and weight reduction before or during pregnancy on maternal and neonatal outcomes. Our review will highlight current evidence gaps and generate meaningful and translatable avenues for research in the area of interventional strategies for maternal obesity. 

### 1.1. Effects of Maternal Obesity on Maternal Outcomes

#### 1.1.1. Rodent Studies

**Figure 1:**
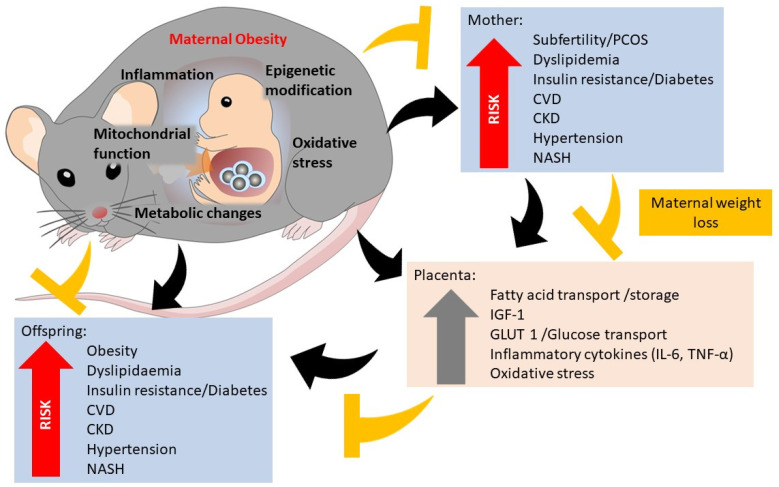
Mechanisms of maternal obesity on maternal outcomes, foetal programming and placental changes (black arrows). Potential pathways for maternal weight loss to reduce maternal offspring risk (yellow arrows). CKD, chronic kidney disease. CVD, cardiovascular disease. NASH, non-alcoholic steatohepatitis. PCOS, polycystic ovarian syndrome. IGF-1, insulin, such as growth factor-1. GLUT-1, glucose transporter-1. IL-6, interleukin-6. TNF-α, tumour necrosis factor-α.

Rodent studies have contributed to the growing body of evidence that exposure to a maternal obesogenic environment has deleterious effects on maternal health. Maternal obesity is associated with reduced fertility [8,9] and increased spontaneous abortion/foetal reabsorption [10,11]. Maternal obesity has been shown to increase maternal insulin resistance and diabetes [12], together with hypertension and concomitant autonomic nervous system dysregulation [13]. Maternal obesity mediates pregnancy-related cardiac hypertrophy with increased left ventricular mass. Persistent postpartum effects of maternal obesity include cardiac hypertrophy and cardiac remodelling, suggesting that the combined effects of pregnancy and obesity are detrimental to cardiovascular outcomes [14,15]. In susceptible genetically-predisposed rats, HFD feeding during pregnancy and lactation leads to obesity and hepatic steatosis in the mothers. HFD-fed mice, in conjunction with injected soluble fms-like tyrosine kinase-1 (SFlt-1) have been used to model maternal pre-eclampsia [16], with maternal hypertension demonstrated (Figure 1). 

High saturated fatty acid (SFA) intake in maternal mice has been shown to be deleterious for total cholesterol and LDL-cholesterol levels [17], which in turn can enhance the susceptibility to oxidative stress and the development of atherosclerosis [18]. Further, it is known that lipids can act as signalling molecules and transcriptional activators, especially in the liver [19]. SFAs can bind to nuclear receptors involved in lipid homeostasis pathways and induce lipid droplet accumulation [20]. Markers of inflammation have been seen in both adipose tissue and the liver of obese rodents [21], including Tumour Necrosis Factor-α, chemokine receptor-2, monocyte chemoattractant protein-1, and toll-like receptor-4 [22,23,24]. Circulating proinflammatory cytokines and free fatty acids (FFA’s) can further lead to insulin resistance [21,25]. Such findings demonstrate the multifaceted yet intersecting pathways of obesity-induced metabolic dysfunction in maternal physiology. 

Abnormal placentation has been observed in rodent models of obesity. Placenta from HFD-fed obese mouse dams show impaired placental development and vascularization, which may mediate foetal growth changes through the reduced transfer of nutrients across the placenta [26]. Chronic inflammation has also been demonstrated in the placenta of obese dams, with an upregulation of inflammatory cytokines [27,28,29,30,31]. There is consistent evidence that maternal obesity is associated with reduced uterine and spiral artery remodelling, resulting in placental insufficiency [31,32,33,34,35]. Epigenetic processes, such as DNA methylation and histone modification, which induce hereditable changes in genome expression without altering gene sequence, are emerging as an important mechanism for such foetal programming [36]. Changes in the expression of epigenetic machinery genes in term placentas have also been demonstrated, with altered gene expression associated with increased transplacental glucose transfer, foetal growth and placental glycogen, and lipid storage [37,38,39,40,41,42]. The upregulation of placental nutrient transporters such as glucose transporter (GLUT) 1 and 3 and large neutral amino acid transporter 1 (LAT1) have been consistently found in the placenta of obese dams [43,44]. Furthermore, placental insulin, growth factor, and leptin signalling appear to upregulate placental mechanistic target of rapamycin (mTOR) activity [45], which positively correlates with foetal growth in animal models [46], illuminating its role as a placental nutrient sensor [47] (Figure 1). 

#### 1.1.2. Human Studies

Similar to the findings in rodent studies, the pathophysiologic changes associated with obesity in human pregnancy include the upregulation of systemic inflammation, hormonal aberrations, and immunologic dysregulation [30,48,49,50,51,52,53,54,55,56]. Further, foetal overgrowth and macrosomia, as seen in rodent models, is a well-defined consequence of maternal obesity, well supported in the literature and clinical practice [57]. These human–rodent correlations further demonstrate the utility of mouse models of obesity to interrogate the cellular mechanisms of obesity in pregnancy. 

Maternal obesity has effects on human placental physiology. The upregulation of inflammation occurs in the placentas of obese women, and increasing inflammatory markers are detected in the foetus [30]. Increased adipokines and cytokine release are seen in severe obesity, with impacts on foetal development [58,59]. Maternal insulin stimulates placental glucose transfer to the foetus via GLUT4, facilitating glucose transfer to the foetus; hence hyperinsulinaemia has a key role in foetal overgrowth [60]. Maternal obesity alters placental metabolism, leading to increased fatty acid oxidation, further exacerbating insulin resistance and high free fatty acid concentrations found in human foetal blood [61,62,63]. 

### 1.2. Effects of Maternal Obesity on Foetal and Offspring Outcomes

#### 1.2.1. Rodent Studies

Overwhelmingly, rodent studies show foetal overgrowth as a major consequence of maternal obesity [43,44,45,64,65]. Higher perinatal weights have been observed from as early as postnatal day 1, with faster weight gain, abdominal adiposity, and circulating cholesterol levels thereafter, once further challenged with an HFD [66]. Maternal obesity has been shown to increase pryoptosis and apoptosis in foetal kidneys, with decreased antioxidants such as superoxide dismutase 2 (SOD2) and catalase and increased oxidative stress marker epoxide hydrolase (Ephx) 2, mediated by the Nod-like receptor protein (NLRP) 3 inflammasome pathways [67]. Maternal obesity has also been shown to have teratogenic effects on cardiac development, with an increased risk of abnormal aortic valve development, endothelial dysfunction, and abnormal hemodynamic function in offspring [68]. Neural development in the developing foetus has also demonstrated alterations in maternal obesity, with hippocampal changes seen by day 17 of gestation [69]. Increased foetal resorption has also been demonstrated in maternal obesity models [69,70]. Failure to thrive and early mortality in the first few days of life are more common in the neonate of obese versus lean dams [71]. 

In comparison to the known mechanisms and effects of maternal obesity on perinatal complications for the mother and the neonate, the developmental influences of maternal obesity on the long-term health of the offspring are less well established, especially in human studies. Rodent studies have been helpful in establishing the growing body of evidence that a maternal obesogenic environment in pregnancy is a determinant of metabolic dysregulation in the offspring that lasts a lifetime. 

A large accumulating body of evidence overwhelmingly supports the concept that maternal obesity programs the development of metabolic disease and obesity in the offspring. Offspring exposed to maternal obesity, fed a hypercaloric diet, show a greater propensity to non-alcoholic liver disease [72]. Adult female offspring of rats fed an HFD through gestation demonstrate raised blood pressure, with a blunting of endothelium-dependent relaxation to acetylcholine [73]. Further, the offspring of obese mothers demonstrate elevated plasma triglyceride and reduced plasma high-density lipoprotein cholesterol levels, with abnormal fatty acid composition in the aorta [74]. The brown adipose tissue metabolism in 16-week-old male offspring of obese dams has also been shown to have a disordered function, with the increased activation of DNA methylation of the genes involved in fatty acid oxidation, thermogenesis and impaired brown adipose tissue structure [75]. Rodent models have clearly demonstrated the detrimental effect of maternal obesity on transgenerational cardiovascular risk. Cardiac remodelling with left ventricular hypertrophy and hypertension has been demonstrated [16,76]. Reduced bone density and the dysregulation of the trabecular architecture have also been noted [77].

Our research team has previously shown that the offspring of obese dams have sustained upregulation of oxidative stress pathways in the kidneys of male adult offspring [78,79]. The mechanisms underpinning the association of maternal obesity with the increased propensity of offspring towards the development of obesity, diabetes, heart disease, and other features of the metabolic syndrome in adulthood are known to involve key regulatory pathways, namely inflammation, oxidative stress, and lipid metabolism dysregulation [80]. 

Maternal obesity affects offspring subfertility. In the male offspring of obese dams, sperm quality and function are impaired, with deficiencies in sperm mitochondrial function and a higher number of abnormal metaphase and greater reactive oxygen species [81]. Similarly, oocytes from successive generations of maternal obesity exposure have been shown to have lower mitochondrial mass and function compared to oocytes from offspring of control mothers, suggesting significant effects on both female and male fertility [82]. 

The programming effects of maternal obesity on the offspring are potentiated by postnatal diet-induced obesity [80]. Our previous rodent studies, and others, have shown that being born to an obese mother significantly potentiates the deleterious effects of an obesogenic diet, yielding reduced glucose tolerance, exaggerated insulin resistance, hepatic steatosis, and renal fibrosis [78,83,84,85,86]. Interestingly, offspring have also been shown to develop hyperphagia [77].

The impact of developmental programming due to maternal obesity differs by sex of the offspring in rodent models. Male offspring, compared to female offspring, show greater body weight, retroperitoneal fat mass, liver mass, plasma leptin levels, and impaired glucose tolerance [87,88]. Furthermore, male offspring have decreased islet number and secretion at postnatal day 21 compared to female offspring [89] (Figure 1).

#### 1.2.2. Human Studies Supporting the Effect of Maternal Obesity on the Offspring

Discerning the impact of genetic disposition, postnatal environment, and foetal programming on offspring outcomes is problematic in human studies. Population-level data suggest that the adult offspring of mothers with obesity have a greater risk of premature death, mainly driven by cardiovascular disease [90]. Higher pre-pregnancy maternal body weight is shown to be associated with greater childhood adiposity and fat distribution [91,92,93]. Gestational weight gain is a critical parameter for maternal health, associated with an increased risk of macrosomia, pre-eclampsia, and maternal obesity post-partum [94]. Further gestational weight gain has been shown to be an independent predictor of total adiposity and body fat distribution in offspring during infancy [95,96]. This maternal obesogenic environment is associated with higher blood pressure, impaired insulin sensitivity, and adverse lipid profiles [97,98,99,100,101]. Epigenetic gene regulation, namely, the altered methylation status of nuclear receptor RXRA, is associated with maternal obesity and childhood adiposity [102]. Data from the Dutch famine demonstrated changes in DNA methylation with increased methylation changes in leptin at key intervals of exposure, namely periconception. This contrasted with exposure in late gestation, where no discernible differences in methylation were seen [103]. Further, Sletner et al. showed that the higher methylation that occurs at placental leptin CpG sites was related to maternal glucose and lipid levels [104]. 

### 1.3. Diet Modulation in Pregnancy and Its Impact on Obesity-Related Maternal and Perinatal Outcomes 

#### 1.3.1. Rodent Studies

Interventional strategies for obesity during pregnancy have the potential to influence health outcomes for both the mother and the offspring. However, few studies have investigated the effect of interventional strategies for weight modulation during pregnancy in rodent models. One study utilised a modified fatty acid composition diet enriched with medium-chain fatty acids and decreased polyunsaturated fats to demonstrate reduced maternal body weight, adipocyte size, and hepatic fat accumulation compared to unmodified HFD fed mice. Moreover, there was a trend toward lower fasting glucose, insulin, and leptin concentrations in the mothers [105]. Fish oil supplementation, used in a rat model of maternal obesity, seemed to ameliorate obesity and insulin resistance, and male offspring had reduced adiposity, hepatic inflammation, and fat deposition in adulthood [106]. Female offspring were not included in this study. 

Increased physical activity in the form of voluntary exercise during pregnancy improves maternal insulin and glucose sensitivity, though this did not translate to reduced body weight, duration of gestation, litter size, or neonatal weight [107]. Importantly the offspring of obese dams had lower body weights and higher glucose uptake into muscles, with lower serum insulin levels compared with the offspring of lean dams [107]. The combined use of diet and exercise also showed beneficial effects on triglyceride levels, glucose and insulin levels, and oxidative stress markers in offspring [108]. 

#### 1.3.2. Human Studies

Studies on pregnant women intending to reduce obesity-related adverse events by limiting gestational weight gain have shown only modest effects. The LIMIT trial was a large study of supervised lifestyle modification in pregnancy involving 2152 women and 2142 liveborn infants. Neither improved maternal outcomes of pregnancy nor reduced neonatal morbidity, including large for gestational age, were seen [5]. UPBEAT was a randomised controlled trial utilising a behavioural intervention in pregnancy to positively impact diet and exercise modification, and it did not show reductions in gestational diabetes mellitus incidence nor the altered incidence of large-for-gestational-age infants, pre-eclampsia, or other pregnancy outcomes [109]. Disappointingly, studies investigating diet change over the course of human pregnancy, intended to limit gestational weight gain, have not demonstrated improved perinatal outcomes, including birth weight and neonatal hypoglycaemia [110]. This may be due to a lack of sustained diet and behavioural modification, the impact of hyperphagia of pregnancy, or a lack of homeostatic resetting with respect to weight. Further, the effect of pre-gestation obesity programming on oocytes has also not been examined and may form a further mechanism by which gestational intervention is ineffective. Whatever the underlying cause, it would seem that in human studies, weight modulation once pregnancy ensues is too little, too late.

### 1.4. Pre-Pregnancy Weight Reduction and Its Impact on Obesity-Related Maternal, Perinatal and Offspring Outcomes

#### 1.4.1. Rodent Studies

There are few studies that have investigated the benefits of pre-pregnancy weight modulation on maternal and offspring health in rodent models of maternal obesity. These studies include interventional strategies with diet modification, dietary supplements, pharmacological agents, and surgery.

Switching the rodent diet from HFD to chow in the preconception period results in progressive improvements in insulin sensitivity, glucose tolerance, and serum insulin over the course of 4 weeks of diet change. Further, cholesterol levels also improved [111,112]. There are benefits for fertility seen after 6 weeks of sustained weight loss, with notable changes in oocyte mitochondrial ultrastructure and improved reactive oxidative stress levels [111]. Maternal diet change prior to pregnancy has also been shown to reduce hepatic steatosis in obese pregnant dams [113]. A follow-up study further showed that the offspring of obese dams had greater adipocyte hypertrophy, with increased macrophage inflation and enhanced cytokine gene expression into adipose tissue, compared to those that switched diets [114]. Treatment with oligofructose led to significantly lower fasting glucose levels in the offspring with a variable effect on offspring body weight [115].

An interesting study that examined the impact of maternal exercise on perinatal outcomes in mice found that exercise throughout the pregestational and gestational periods, rather than pregnancy alone, was most efficacious for improving glucose tolerance and peripheral insulin sensitivity [116]. Such effects were independent of maternal body weight, as training of dams did not significantly affect maternal body weight. Further, there was no difference in conception rates or litter size [116]. 

Pre-pregnancy pharmacological interventions are limited. Dennison et al. investigated preconception use of probiotics alone or in combination with a dipeptidyl peptidase 4 inhibitor (DPP-4 i), sitagliptin, with or without probiotics, and found reduced maternal body weight, improved insulin sensitization and outcomes of pregnancy in obese mice but not improved fertility [115]. 

Our recent study showed that the use of the glucose-like peptide-1 (GLP-1) agonist, liraglutide, in the preconception period is an effective interventional strategy to reduce maternal body weight, improve maternal glucose tolerance and lipid metabolism, and fertility rates were improved in obese mice treated with pre-pregnancy liraglutide [112]. Interestingly, gestational weight gain following the cessation of liraglutide in pregnancy and in the absence of diet change led to ‘’catch up’’ gestational weight gain, such that by the end of pregnancy, mice treated with liraglutide pre-pregnancy showed similar glucose tolerance and body composition as obese mice receiving placebo injections prior to pregnancy [112]. 

The use of preconception bariatric surgery to facilitate rapid and significant weight loss has been examined in HFD-fed rodent models of maternal obesity. A study using vertical sleeve gastrectomy, involving an 80% excision of the stomach, resulted in significantly reduced caloric intake, adiposity, circulating lipids, and improved glucose tolerance in the dams and their reproductive cycle normalized inferring better fertility outcomes. Offspring, however, showed evidence of growth restriction, with greater levels of glucose intolerance and increased adiposity than the offspring of lean mothers or obese mothers who underwent sham surgery [117]. Similar effects were also seen in the offspring of dams that received Roux-en-Y gastric bypass surgery before pregnancy, a procedure known to involve a restrictive and malabsorptive component [118]. 

Overall, preconception diet, exercise and pharmacotherapy for weight loss show promising signs of benefits for maternal and neonatal health; however, significant weight loss with surgical intervention appears to be too drastic with potentially adverse outcomes for the offspring. Importantly, no studies to date have examined the impact of pre-pregnancy intervention for maternal obesity on the long-term health of the offspring.

#### 1.4.2. Human Studies

The benefits of preconception weight loss have been explored in few human studies. Hypocaloric diets in the preconception period can induce weight loss and, therefore, subsequently improve fertility [119]. The Pre-babe Pilot study investigating the use of caloric restriction with meal replacements was acceptable to patients and resulted in significant pre-pregnancy weight loss compared to diet alone; however, the benefits for fertility, maternal, and foetal outcomes have not yet been reported [120]. A prospective randomised controlled study utilising a 12-week weight loss program showed significant weight loss, increased sex-hormone-binding globulin, and ovulation, but without any changes in mean LH or FSH concentrations [121]. Perinatal outcomes were not assessed in this study. A similar study, looking at weight loss over 6 months in women with polycystic ovarian syndrome, also demonstrated improved conception rates [122]. In all of the studies utilising dietary and exercise intervention advice, pre-pregnancy weight loss has been modest (2–3%), and the study design was limited by blinding bias, which is inevitable in a study predominantly involving lifestyle intervention advice in humans. Fertility guidelines do recommend weight loss in the preconception period, on the strength of these studies [123]. Overall, however, the benefit of recommendations for lifestyle advice is still unclear, with further studies required [124]. 

Bariatric surgery before pregnancy is an effective approach for achieving pre-pregnancy weight loss and improved fertility with a lower incidence of miscarriage or congenital malformation in women with severe obesity. However, weight loss surgery can lead to vitamin deficiencies, preterm delivery, and small-for-gestational-age outcomes if pregnancy ensues within 12 months of surgery [125,126]. As a result, recommendations for pregnancy after bariatric surgery suggest delaying conception until weight loss has stabilized post-operatively, which can take up to 12 months [127]. For the majority of women with obesity, bariatric surgery is neither desired nor indicated, and hence dietary modification and pharmacotherapy remain the most practical alternatives. 

## 2. Future Directions

The research to date has uncovered many avenues for further investigation. There is still much to uncover about the mechanisms underpinning foetal programming. The role of oxidative stress and meta-inflammation (the combination of low-grade chronic inflammation and metabolic dysfunction observed in diabetes and obesity [128]) in offspring disease development is fascinating and still to be fully appreciated. Further, the impact of maternal obesity at the maternal-foetal interface and the role of the placenta in modulating the environmental impact of the maternal environment requires further mechanistic probing. The role of epigenetic modification in transgenerational disease risk is only just being uncovered. There is still much to learn about exposure windows in utero (e.g., early, mid vs. late gestation), as well as methods of altering adverse epigenetic signatures. Inter-individual variation in how the genome and epigenome are modulated by maternal perturbations requires further exploration. 

The possible avenues for intervention are numerous and worthy of further investigation. Either pre- or interpregnancy weight-loss interventions are still not demonstrably effective and require further study. The role of neural mechanisms, hormonal pathways, and gut absorption are a few possibilities for targeted intervention to reduce the impact of obesity. Postnatal interventions for offspring could also be considered to reduce the chronic disease burden in the offspring. Drug development, including more potent GLP-1 receptor agonists, is revolutionizing obesity management and has exciting implications for women of reproductive age. The prevalence of surgical procedures is also increasing, with implications for the health of both the mother and the offspring. These surgical procedures, and emerging options such as Endobarrier © [129], require further investigation into best usage, as well as specific health outcomes. 

## 3. Conclusions

In view of the growing body of evidence regarding the transgenerational consequences of maternal obesity, it is paramount that efforts are directed to discover ways to ameliorate or mitigate the impacts of maternal obesity on adverse foetal programming. Overall, the evidence for the efficacy of weight modulation strategies during pregnancy is disappointing, and the preconception period is emerging as a time where meaningful gains may be made. Devising effective pre-pregnancy strategies, utilising diet modification, pharmacotherapy, or surgical intervention, are necessary to halt the transgenerational propagation of obesity. The utilisation of animal models to facilitate mechanistic and therapeutic discovery continues to be central to these discoveries. The ease of modelling, the reproductive potential, and the translational capacity of rodent models make them ideally placed to facilitate translation from bench to bedside discovery. Increasing the urgency of women with obesity of reproductive age to consider their fertility potential, plan pregnancy, and utilise intervention to improve pregnancy outcomes for themselves and their babies deserves increased public health attention worldwide.
